# Defining Peri-Operative Myocardial Injury during Cardiac Surgery Using High-Sensitivity Troponin T

**DOI:** 10.3390/jcm12134291

**Published:** 2023-06-26

**Authors:** Vikram Sharma, Huili Zheng, Luciano Candilio, Jennifer M. Nicholas, Tim Clayton, Derek M. Yellon, Heerajnarain Bulluck, Derek J. Hausenloy

**Affiliations:** 1 Department of Cardiovascular Medicine, University of Iowa, Iowa City, IA 52242, USA; drvikramsharma@hotmail.com; 2 National Registry of Diseases, Health Promotion Board, Singapore 168937, Singapore; zheng.huili@ktph.com.sg; 3 Department of Cardiology, Royal Free Hospital, London NW3 2QG, UK; luciano2108@hotmail.com; 4 Clinical Trials Unit, Department of Medical Statistics, London School of Hygiene & Tropical Medicine, London WC1E 7HT, UK; jennifer.nicholas@lshtm.ac.uk (J.M.N.); tim.clayton@lshtm.ac.uk (T.C.); 5 The Hatter Cardiovascular Institute, University College London, London WC1E 6HX, UK; d.yellon@ucl.ac.uk; 6 Leeds Institute of Cardiovascular and Metabolic Medicine, University of Leeds, Leeds LS2 9JT, UK; h.bulluck@gmail.com; 7 Department of Cardiology, Leeds General Infirmary, Leeds Teaching Hospitals NHS Trust, Leeds LS1 3EX, UK; 8 Cardiovascular & Metabolic Disorders Program, Duke-National University of Singapore Medical School, Singapore 169857, Singapore; 9 National Heart Research Institute Singapore, National Heart Centre, Singapore 169609, Singapore; 10 Yong Loo Lin School of Medicine, National University Singapore, Singapore 117597, Singapore; 11 Cardiovascular Research Center, College of Medical and Health Sciences, Asia University, Taichung 41354, Taiwan

**Keywords:** peri-operative myocardial injury, high-sensitivity troponin, coronary artery bypass graft surgery, mortality

## Abstract

Objective: Cut-offs for high-sensitivity troponin (hs-Tn) elevations to define prognostically significant peri-operative myocardial injury (PMI) in cardiac surgery is not well-established. We evaluated the associations between peri-operative high-sensitivity troponin T (hs-TnT) elevations and 1-year all-cause mortality in patients undergoing cardiac surgery. Methods: The prognostic significance of baseline hs-TnT and various thresholds for post-operative hs-TnT elevation at different time-points on 1-year all-cause mortality following cardiac surgery were assessed after adjusting for baseline hs-TnT and EuroSCORE in a post-hoc analysis of the ERICCA trial. Results: 1206 patients met the inclusion criteria. Baseline elevation in hs-TnT >x1 99th percentile upper reference limit (URL) was significantly associated with 1-year all-cause mortality (adjusted hazard ratio 1.90, 95% confidence interval 1.15–3.13). In the subgroup with normal baseline hs-TnT (*n* = 517), elevation in hs-TnT at all post-operative time points was associated with higher 1-year mortality, reaching statistical significance for elevations above: ≥100 × URL at 6 h; ≥50 × URL at 12 and 24 h; ≥35 × URL at 48 h; and ≥30 × URL at 72 h post-surgery. Elevation in hs-TnT at 24 h ≥ 50 × URL had the optimal sensitivity and specificity (73% and 75% respectively). When the whole cohort of patients was analysed, including those with abnormal baseline hs-TnT (up to 10 × URL), the same threshold had optimal sensitivity and specificity (66% and 70%). Conclusions: Both baseline and post-operative hs-TnT elevations are independently associated with 1-year all-cause mortality in patients undergoing cardiac surgery. The optimal threshold to define a prognostically significant PMI in our study was ≥50 × URL elevation in hs-TnT at 24 h.

## 1. Introduction

Peri-operative myocardial injury (PMI) in the setting of cardiac surgery is defined as an elevation in biomarkers of myocardial injury irrespective of any additional evidence of myocardial ischemia/infarction. It is increasingly being recognised as an important determinant of post-operative mortality [1,2]. The causes of PMI in cardiac surgery are diverse, including trauma of the myocardium from surgical manipulation, inadequate cardioprotection during cardiopulmonary bypass, mismatch in myocardial oxygen demand-supply due hemodynamic instability or from other perioperative complications [1]. 

The universal definition consensus document [2] defines acute myocardial infarction in the setting of coronary artery bypass graft surgery (CABG) (Type 5 myocardial infarction) as elevation of cardiac troponin (cTn) values >10 times the 99th percentile upper reference limit (URL) in patients with normal baseline cTn values. However, clear universal definitions of PMI in cardiac surgery have yet to be established, particularly using the high sensitivity troponin (hs-Tn) assays [2,3,4]. Several studies have recently shown that post-operative elevation in high sensitivity troponin (hs-Tn) assay was associated with poorer outcomes following cardiac surgery [3,4,5,6]. Of note, in patient without myocardial infarction post-CABG, elevations in hs-Tn have been shown to be much higher than the threshold for type 5 myocardial infarction definition using conventional Tn essays [7,8]. None of these studies performed an in-depth analysis of the impact of hs-Tn elevations post-operatively, at serial time points ranging from very early (6 h) and up to 72 h on 1-year all-cause mortality. 

Therefore, we aimed to investigate the association between baseline (pre-operative), as well as post-operative elevations in hs-TnT on 1-year all-cause mortality in stable patients undergoing cardiac surgery (CABG ± valve surgery). In particular, we aimed to provide optimal cut-offs for hs-TnT elevations above the 99th percentile URL at different time points after cardiac surgery that would define prognostically significant PMI, irrespective of any electrocardiographic or imaging evidence of myocardial ischemia or infarction.

## 2. Methods

This was a post-hoc analysis from the previously published Effect of Remote Ischemic Preconditioning on Clinical Outcomes in Patient Undergoing Coronary Artery Bypass Graft Surgery (ERICCA trial [9,10,11]. The ERICCA trial [9] was a multicenter (conducted at 30 UK sites) randomized controlled trial of 1612 on-pump CABG ± valve surgery patients which failed to show any benefit of remote ischemic preconditioning on clinical outcomes. Only patients with an additive European System for Cardiac Operative Risk Evaluation (EuroSCORE) of 5 or higher were included in this trial. The study was approved by the National Health Service Research Ethics Committee (East London 3–REC reference number: 10/H0701/111) and was conducted in accordance with the principles of Good Clinical Practice under the oversight of University College London Hospital. All participants provided written informed consent. 

The primary outcome of this analysis was 1-year all-cause mortality. Hs-TnT was measured at baseline, 6-, 12-, 24-, 48- and 72-h post-surgery using the one-step immunoassay based on electrochemiluminescence technology (Elecsys 2010, Roche, Basel, Switzerland). The upper reference range was ≤14 ng/L. 

**Figure 1:**
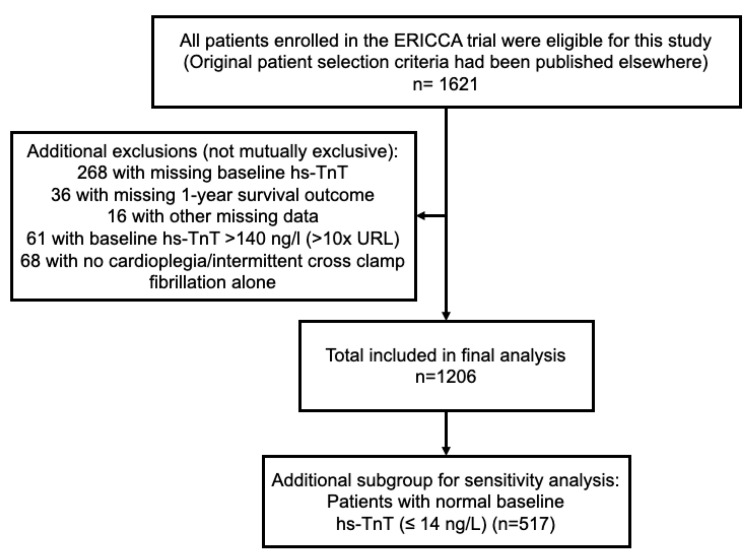
Flow diagram showing criteria for patient selection for the study from the ERICCA trial cohort.

For the purpose of this post-hoc analysis, we excluded patients who had a baseline hs-TnT elevation >10 × URL (to exclude those with recent myocardial infarction or significant baseline hs-TnT rise from other secondary causes); those with missing baseline troponin levels; those with missing 1-year survival data; those who did not receive cardioplegia for cardioprotection during surgery (since lack of cardioplegia can significantly affect the extent of troponin elevation following cardiac surgery and thereby influence subsequent clinical outcomes). We also excluded patients who had missing data on other key variables (Figure 1). 

### Statistical Analysis

Missing values of hs-TnT at specific time points post-surgery were imputed on the natural logarithm scale, using Gaussian normal regression multiple imputations on the patients meeting the inclusion criteria by chain equations with twenty imputed datasets. Nelson-Aalen estimates of the cumulative baseline hazard at the time of event/censoring, baseline troponin, EuroSCORE and 1-year all-cause mortality were included in the imputation model. The other available variables were not included in the imputation model as most of them are included in the EuroSCORE. 

Using 1-year all-cause mortality as the outcome, the area under the receiver operating characteristics curve (AUC) for hs-TnT elevation was used to identify the threshold at each post-operative time point with the maximum sum of sensitivity and specificity, without adjusting for any covariate in the subgroup of patients with normal baseline hs-TnT (≤14 ng/L) (Supplementary Figure S1). These thresholds were then applied on the whole cohort, including those with >×1 URL elevations in hs-TnT. The HR for 1-year all-cause mortality for different hs-TnT elevations at various time points were estimated using Cox regression, adjusted for EuroSCORE and baseline hs-TnT. 

The impact of baseline hs-TnT elevation on left ventricular systolic function (represented by left ventricular ejection fraction (LVEF)) was evaluated by stratifying the entire cohort based on baseline hs-TnT (normal ≤1 × URL versus elevated >1 × URL) and ejection fraction (divided into three groups: severely reduced LVEF < 30%, moderately reduced LVEF 30–50%, and normal LVEF > 50%). All statistical analyses for the study were carried out using STATA SE version 13.

## 3. Results

From the total cohort, 1206 patients met the inclusion criteria (Figure 1). The characteristics for these patients are summarized in Table 1. The median age of the study population was 77 years and the majority (71%) were male. 94% of the patients in the study were Caucasian. Half of the patients (52%) had valve surgery in addition to CABG. The median EuroSCORE was 6 (IQR 5–7). 12 (1%) patients died within 72 h post-surgery, while 90 (7.5%) patients died within 1 year. 

**Figure 2:**
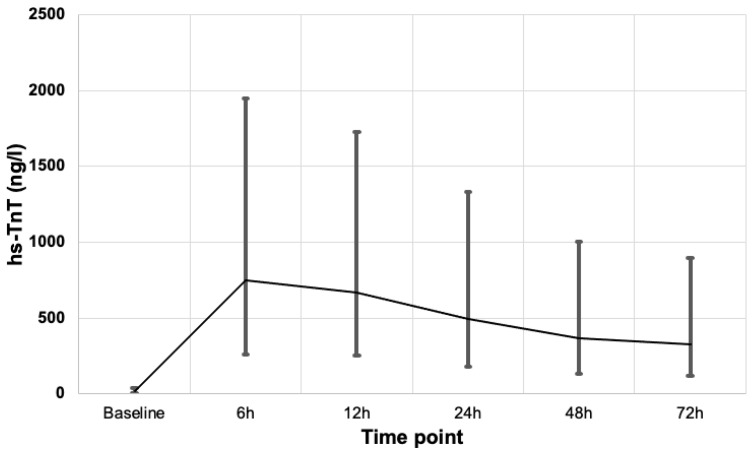
Median hs-TnT at various time points after surgery; vertical lines are the interquartile ranges.

Figure 2 shows the median hs-TnT elevation at various time-points after cardiac surgery and their respective interquartile ranges and post-operative hs-TnT peaked at 6 h in the cohort. Early peaks in hs-TnT elevation were common, occurring at 6 h in 64% of the patients and at 12 h in 21% of the patients. In comparison only 4% of the patients had hs-TnT elevation peaking at 48 h. Notably, the proportion of patients who had increased 1-year mortality was much lower in patients with an early peak at 6–12 h (6–10%) compared to those with a later peak (20% in patients with hs-TnT peaked at 48 h).

### 3.1. Baseline Tn-TnT Elevation and 1-Year All-Cause Mortality

**Figure 3:**
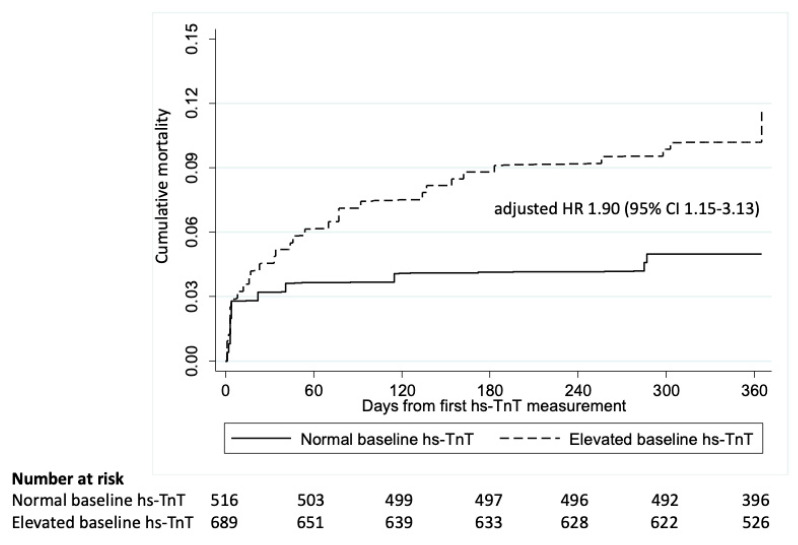
Kaplan Meier curve of patients with normal baseline hs-TnT compared to those with elevated baseline hs-TnT in the whole cohort.

517 patients (43%) had baseline hs-TnT levels <×1 URL. Baseline elevation in hs-TnT >1 × URL was significantly associated with 1-year all-cause mortality after adjusting for EuroSCORE (adjusted HR 1.90, 95% CI 1.15–3.13) (Figure 3), when compared with those with normal baseline hs-TnT.

### 3.2. Post-Operative Elevations in hs-TnT and 1-Year All-Cause Mortality in Patients with Normal Baseline hs-TnT (N = 517)

Post-operative hs-TnT elevation was significantly associated with 1-year all-cause mortality in the cohort of patients with normal baseline hs-TnT (Table 2). Elevations at all time points and all thresholds showed an association with greater mortality, reaching statistical significance for elevations: ≥100 × URL at 6 h; ≥50 × URL at 12 h and 24 h, respectively; ≥35 × URL at 48 h; and ≥30 × URL at 72 h (Table 2). 

The sensitivity and specificity for each threshold at the different time points and the AUC are provided in Table 2. Threshold ≥70 × URL in hs-TnT at 48 h was the most specific (92%) and the ≥35 × URL at 12 h was the most sensitive (95%). However, the AUCs were similar for the 4 time points. The threshold that provided the optimal sensitivity and specificity (by Youden Index) was ≥50 × URL elevation in post-operative hs-TnT at 24 h (sensitivity: 73%; specificity: 75%).

### 3.3. Post-Operative Elevations in hs-TnT and 1-Year All-Cause Mortality in Patients with Elevated hs-TnT (N = 689)

In the subgroup of patients with elevated baseline hs-TnT, post-operative hs-TnT elevation was also significantly associated with 1-year all-cause mortality in the cohort of patients with normal baseline hs-TnT (Table 3). Elevations at all time points and all thresholds showed an association with greater mortality, reaching statistical significance for elevations above: ≥100 × URL at 6 h; ≥50 × URL at 12 h and 24 h, respectively; ≥35 × URL at 48 h; and ≥30 × URL at 72 h (Table 3). These thresholds at the specific time points were identical to those that reached statistical significance for the subgroup of patients with normal baseline hs-TnT.

The sensitivity and specificity for each threshold at the different time points and the AUC are provided in Table 3. Threshold ≥70 × URL in hs-TnT at 48 h was the most specific (86%) and the ≥35 × URL at 12 h was the most sensitive (81%). However, the AUCs were similar for the 4 time points. The threshold that provided the optimal sensitivity and specificity (by Youden Index) was ≥50 × URL elevation in post-operative hs-TnT at 24 h (sensitivity: 65%; specificity: 67%) and these where lower than in patients with normal baseline hs-TnT.

### 3.4. Post-Operative Elevations in hs-TnT and 1-Year All-Cause Mortality in the Whole Cohort (N = 1206)

The minimum threshold for a statistically significant prognostic elevation in post-operative hs-TnT in the whole cohort of patients, including those with baseline elevation in hs-TnT (up to 10 × URL), were identical (> 100 × URL at 6 h; ≥50 × URL at 12 h and 24 h, respectively; ≥35 × URL at 48 h; and ≥30 × URL at 72 h) to the ones obtained in the cohort with normal baseline hs-TnT. The threshold that provided the optimal sensitivity and specificity was also ≥50 × URL elevation in post-operative hs-TnT at 24 h (sensitivity: 66%; specificity: 70%).

**Figure 4:**
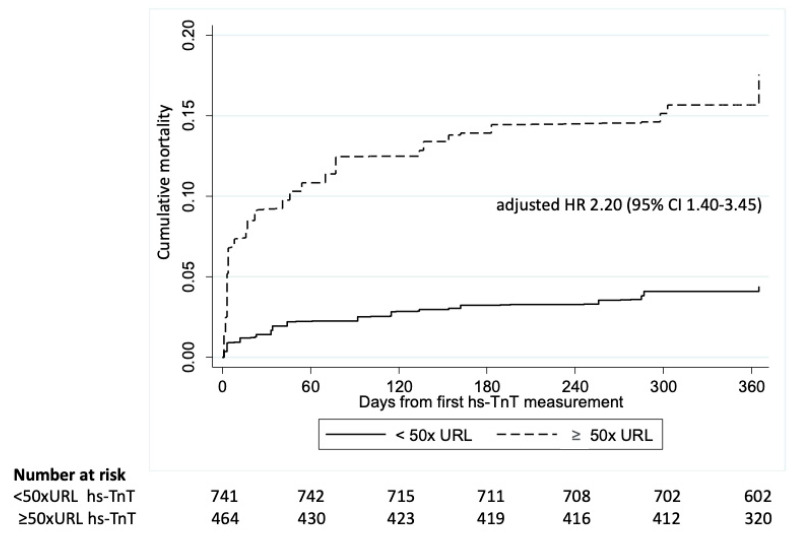
Kaplan Meier curve of patients with <50 × URL elevation in post-operative hs-TnT at 48-h post operation compared to those with ≥50 × URL elevation in post-operative hs-TnT in the whole cohort.

Post-operative elevation in hs-TnT ≥50 × URL was significantly associated with 1-year all-cause mortality after adjusting for EuroSCORE (adjusted HR 2.20, 95% CI 1.40-3.45) (Figure 4), when compared with those with post-operative elevation in hs-TnT <50 × URL.

### 3.5. Stratification by Left Ventricular Ejection Fraction (LVEF)

Among patients with normal LVEF, elevated post-operative hs-TnT was associated with increased 1-year all-cause mortality, reaching statistical significance for elevations ≥100 × URL at 6 h, ≥50 × URL at 12 h and 24 h, ≥35 × URL at 48 h, and ≥30 × URL at 72 h (Supplementary Table S1). The threshold with the optimal sensitivity and specificity was also ≥50 × URL elevation at 24 h for patients with normal LVEF (sensitivity 62%; specificity 69%).

Among patients with moderately reduced EF, elevated post-operative hs-TnT was similarly associated with increased 1-year all-cause mortality, reaching statistical significance for elevations ≥70 × URL at 24 h, ≥50 × URL at 48 h, and ≥35 × URL at 72 h (Supplementary Table S2). Compared to those with normal LVEF, higher hs-TnT elevations at each time point, especially for earlier time points, are required for patients with moderately reduced LVEF to reach statistical significance. 

Among patients with severely reduced EF, elevated post-operative hs-TnT was likewise associated with higher 1-year all-cause mortality, reaching statistical significance for elevations ≥100 × URL at 6 h, ≥70 × URL at 48 h, and ≥30 × URL at 72 h (Supplementary Table S3). Compared to those with normal LVEF, statistical significance was reached at later time points for patients with severely reduced LVEF.

## 4. Discussion

Our study shows that both baseline as well as post-operative elevations in hs-TnT among patients undergoing CABG ± valve surgery were associated with higher 1-year all-cause mortality. This association was evident as early as 6 h after surgery. The minimum prognostic threshold of hs-TnT threshold was progressively lower with later time points and were as follows: ≥100 × URL elevation at 6 h; ≥50 × URL at 12 h and at 24 h; ≥35 × URL at 48 h; and ≥30 × URL at 72 h, with ≥50 × URL at 24 h providing the optimal sensitivity and specificity. Furthermore, early peak in hs-TnT elevation was common, occurring at 6 h in 64% of the patients and at 12 h in 21% of the patients. Interestingly, the proportion of patients who had increased 1-year mortality was much lower in patients with an early peak at 6–12 h (6–10%) compared to those with a later peak (20% in patients with hs-TnT peaked at 48 h). It may be postulated that later peaks in hs-TnT may carry more prognostic value than an early peak.

The current Universal Definition of Myocardial Infarction (UDMI) proposed a cut-off of >10 × 99th percentile URL elevation in hs-TnT in the first 48 h of CABG in patients with a normal baseline cardiac troponin value to define Type 5 myocardial infarction in the presence of other ECG/angiographic/imaging evidence of myocardial ischemia. However, this cut-off was not defined specifically for hs-TnT. While the Universal Definition task force acknowledged the prognostic importance of “marked” increase in cardiac troponins following cardiac surgery by itself (independent of any additional evidence of myocardial ischemia or infarction), the Universal Definition did not include a specific cut-off for defining prognostically significant myocardial injury in the setting of CABG [2]. In comparison to the UDMI definition, the Society for Cardiovascular Angiography and Interventions (SCAI) and ARC-2 proposed a higher threshold of troponin elevation to define clinically relevant PMI as ≥35 × URL elevation in troponin in presence of additional evidence of coronary ischemia or ≥70 × URL elevation by itself [12,13]. A recent position paper from the ESC joint working group on cardiovascular surgery and cellular biology of the heart, proposed ≥7 × URL elevation in cTnT and ≥20× URL cTnI (non-high sensitivity assays) as the definition of PMI based on a comprehensive analysis of the evidence from prior studies, but acknowledged the lack of data to propose evidence-based definition for PMI in terms of high-sensitivity troponin assays [1]. 

Recent studies including our study highlight some important challenges in being able to define PMI in cardiac surgery due to the differences in the prognostic thresholds based on the high-sensitivity assay used, and the time following cardiac surgery when the troponin is measured. Prior studies have investigated the association between post-operative hs-Tn elevation and mortality as well as other adverse outcomes after cardiac surgery, particularly CABG [3,4]. One of the studies showed that this association was present only when combined with increased pre-operative hs-TnT levels [14]. Post-operative hs-TnT elevation approximately >110 × URL were predictive of adverse outcomes post-cardiac surgery in one study, but this was not specific to a particular time-point after surgery [4]. Another study showed that pre-operative hs-TnT elevation was not associated with in-hospital MACCE (major adverse cardiac or cerebrovascular event) in 203 CABG patients, but 6–12 h post-operative hs-TnT elevation at approximately >60 × URL was associated with increased MACCE [15]. A further study showed that hs-TnT elevation at approximately >30 × URL measured within 24 h of CABG was associated with MACCE and 30-day mortality [16]. These studies demonstrate that the extent of troponin elevation that is prognostically significant varies greatly based on the post-operative time interval and the choice of clinical endpoint. The two most recently published studies by Omran et al. [5] and Devereaux et al. [6] both utilized hs-TnI instead of hs-TnT. Omran et al. [5] showed that an elevation of >500 × URL in hs-TnI within 40 h of cardiac surgery was associated with increased repeat revascularization and that this extent of hs-TnI elevation also predicted 30-day major adverse cardiovascular events as well as all-cause mortality after a median follow-up of 3.1 years in a cohort of 4684 patients undergoing isolated CABG a single center [5]. Of note, the aim of this study was to define the optimal hs-TnI elevation to aid clinical decision making for repeat revascularization rather than defining threshold elevations at different time points associating with post-operative mortality. Devereaux et al. [6] showed in a cohort of 13,862 patients undergoing CABG/AVR, that >218 × URL elevation in hs-TnI at less than day 1 of surgery was associated with 30-day all-cause mortality; and on day 2–3 this threshold was >59 × URL. Although they used hs-TnI rather that hs-TnT and looked at 30-day rather than 1-year all-cause mortality, their day 2–3 threshold of >59 × URL elevation in hs-TnI was similar to the optimal threshold of ≥50 × URL elevation in hs-TnT at 24 h found in our study. Whether this threshold could be used as a surrogate endpoint for a prognostically significant peri-operative myocardial injury post cardiac surgery warrants validation in future studies. Recent metanalyses have shown that hs-TnT elevations are higher that UDMI type 5 myocardial definition post cardiac surgery in patients without myocardial infarction [7,8] and our work build on these findings. Our study has focussed specifically on defining such thresholds, which may be very informative for clinicians in interpreting the prognostic significance of hs-Tn elevation in their patients in the context of recent cardiac surgery. Furthermore, we have showed that such thresholds may be impacted by other clinical factors such as baseline cardiac function but our analysis may have been underpowered. Therefore, the impact of LV function on these prognostic thresholds at different time points after cardiac surgery should be investigated in future studies.

## 5. Limitations

This study is a post-hoc analysis of data from the ERICCA trial, which was not specifically designed to study the PMI thresholds that are prognostically significant. Hence, the trial may not be sufficiently powered to pick up the differences in HR and AUC for various time points. Our study included patients that had CABG ± valve surgery and hence did not exclusively study patients undergoing CABG alone. We excluded patients with a baseline elevation in hs-TnT >10 × URL and these findings would not be applicable to those patients. The study population consisted predominantly of Caucasians and whether ethnicity would have an impact on the threshold in hs-Tn elevation post cardiac surgery warrants further evaluation.

## 6. Conclusions

Baseline elevations in hs-TnT as well as post-operative hs-TnT elevations following CABG ± valve surgery are associated with 1-year all-cause mortality. Post-operative hs-TnT elevation has significant impact on 1-year all-cause mortality, even when patients with up to 10 × URL in baseline hs-TnT levels were included. The optimal threshold to define prognostically significant PMI in our study was ≥50 × URL elevation in hs-TnT at 24 h and this warrants further validation in future studies before it can be used as a surrogate endpoint in clinical trials.

## Figures and Tables

**Table 1:** Baseline characteristics, past medical history and surgery details of patients included in this study (*N* = 1206).

Baseline Characteristics	
**Age in years, median (IQR)**	77 (73–81)
**Sex, *n* (%)**	
Female	350 (29.0)
Male	856 (71.0)
**Ethnicity, *n* (%)**	
Caucasian	1139 (94.4)
Asian	57 (4.7)
Afro-Caribbean	5 (0.4)
Others	5 (0.4)
**Body mass index in kg/m^2^, median (IQR)**	27.2 (24.6–30.4)
**Smoking status, *n* (%)**	
Current	62 (5.1)
Former	707 (58.6)
Never	437 (36.2)
**Systolic blood pressure in mmHg, median (IQR)**	133 (120–147)
**Diastolic blood pressure in mmHg, median (IQR)**	70 (63–79)
**Heart rate in bpm, median (IQR)**	67 (60–76)
**NYHA class, *n* (%)**	
0	172 (14.3)
I	168 (13.9)
II	514 (42.6)
III	329 (27.3)
IV	23 (1.9)
**CCS class, *n* (%)**	
0	361 (29.9)
I	207 (17.2)
II	383 (31.8)
III	171 (14.2)
IV	84 (7.0)
**Left ventricular ejection fraction, *n* (%)**	
>50%	795 (68.7)
30–50%	239 (20.7)
<30%	123 (10.6)
**Past medical history**	
**Diabetes mellitus, *n* (%)**	325 (27.0)
**Hypertension, *n* (%)**	933 (77.4)
**Hypercholesterolemia, *n* (%)**	850 (70.5)
**Atrial fibrillation, *n* (%)**	195 (16.2)
**Myocardial infarction, *n* (%)**	463 (38.4)
**Percutaneous coronary intervention, *n* (%)**	181 (15.0)
**Stroke, n (%)**	136 (11.3)
**Coronary artery bypass graft, *n* (%)**	33 (2.7)
**Peripheral arterial disease, *n* (%)**	90 (7.5)
**Pre-op NGAL in ng/mL, median (IQR)**	173.2 (115.8–253.7)
**Pre-op creatinine in mol/µL, median (IQR)**	90 (76–108)
**EuroSCORE, median (IQR)**	6 (5–7)
**Surgery details**	
**Bypass duration in minutes, median (IQR)**	106 (84–136)
**Cross-clamp duration in minutes, median (IQR)**	71 (51–97)
**Valve surgery, *n* (%)**	621 (51.5)
**Number of grafts, *n* (%)**	
1	254 (21.5)
2	294 (24.9)
3	462 (39.2)
≥4	169 (14.3)
**Troponin T details**	
**Available at 6 h, *n* (%)**	1048 (86.9)
**Available at 12 h, *n* (%)**	1030 (85.4)
**Available at 24 h, *n* (%)**	1063 (88.1)
**Available at 48 h, *n* (%)**	947 (78.5)
**Available at 72 h, *n* (%)**	827 (68.6)

**Table 2:** Performance of hs-TnT elevation at 6, 12, 24, 48 and 72 h after CABG in predicting 1-year all-cause mortality among patients with normal baseline hs-TnT (*N* = 517).

Time Point	×URL	Deaths/N for Patients with hs-TnT above Cut-Off	Deaths/N for Patients with hs-TnT below Cut-Off	Sensitivity	Specificity	Adjusted HR (95% CI) *	AUC (95% CI) *
6 h	50	23/295	4/222	85%	45%	1.54 (0.63–3.79)	0.675 (0.561–0.789)
	70	18/188	10/329	66%	65%	1.58 (0.67–3.71)	0.667 (0.551–0.784)
	100	15/101	13/416	54%	82%	2.86 (1.15–7.10)	0.685 (0.573–0.798)
12 h	35	26/350	1/167	95%	34%	1.66 (0.62–4.47)	0.677 (0.564–0.790)
	50	25/246	2/271	92%	55%	2.71 (1.07–6.87)	0.715 (0.610–0.820)
	70	18/123	9/366	66%	75%	3.33 (1.43–7.74)	0.723 (0.618–0.828)
24 h	35	23/254	4/263	84%	53%	1.76 (0.74–4.20)	0.685 (0.575–0.795)
	50	20/145	7/372	73%	75%	2.99 (1.29–6.90)	0.705 (0.589–0.822)
	70	18/90	9/427	66%	85%	3.87 (1.59–9.40)	0.730 (0.618–0.843)
48 h	35	21/171	6/346	78%	69%	3.47 (1.44–8.33)	0.731 (0.626–0.836)
	50	19/97	9/420	68%	84%	3.96 (1.57–9.95)	0.740 (0.634–0.845)
	70	14/54	14/463	50%	92%	5.03 (1.80–14.01)	0.728 (0.613–0.843)
72 h	30	21/184	7/333	75%	67%	3.49 (1.31–9.30)	0.742 (0.645–0.840)
	35	19/154	9/363	69%	73%	3.23 (1.21–8.59)	0.740 (0.645–0.835)
	50	13/85	15/432	47%	85%	2.18 (0.66–7.15)	0.709 (0.602–0.817)

* Adjusted for baseline hs-TnT and EuroSCORE. URL: upper reference limit; N: total number of patients; HR: hazard ratio; 95% CI: 95% confidence interval; AUC; area under the Receiver Operator Characteristic curve.

**Table 3:** Performance of hs-TnT elevation at 6, 12, 24, 48 and 72 h after CABG in predicting 1-year all-cause mortality among patients with elevated baseline hs-TnT (>1 × URL but <10 × URL) (N = 689).

Time Point	×URL	Deaths/N for Patients with hs-TnT above Cut-Off	Deaths/N for Patients with hs-TnT below Cut-Off	Sensitivity	Specificity	Adjusted HR (95% CI) *	AUC (95% CI) *
6 h	50	54/357	23/332	70	51	1.47 (0.88–2.45)	0.611 (0.537–0.685)
	70	41/242	35/447	54	67	1.54 (0.92–2.56)	0.619 (0.546–0.692)
	100	33/136	44/553	43	83	2.60 (1.52–4.44)	0.656 (0.582–0.729)
12 h	35	62/466	15/223	81	34	1.41 (0.78–2.55)	0.616 (0.541–0.690)
	50	52/338	25/351	68	53	1.71 (1.02–2.88)	0.628 (0.554–0.702)
	70	40/223	37/466	52	70	1.85 (1.11–3.10)	0.640 (0.570–0.711)
24 h	35	54/365	23/324	71	49	1.47 (0.87–2.48)	0.619 (0.546–0.691)
	50	49/254	27/435	65	67	2.03 (1.20–3.42)	0.648 (0.576–0.719)
	70	37/145	39/544	49	83	2.17 (1.26–3.73)	0.657 (0.586–0.727)
48 h	35	50/286	26/403	66	62	2.12 (1.25–3.57)	0.656 (0.584–0.728)
	50	40/183	37/506	52	77	2.22 (1.29–3.81)	0.655 (0.583–0.727)
	70	32/115	45/574	42	86	3.35 (1.91–5.87)	0.688 (0.616–0.760)
72 h	30	51/296	26/393	67	60	2.14 (1.25–3.66)	0.653 (0.580–0.726)
	35	48/259	29/430	62	66	2.15 (1.29–3.58)	0.652 (0.581–0.723)
	50	36/154	40/535	47	81	2.64 (1.54–4.53)	0.657 (0.584–0.731)

* Adjusted for baseline hs-TnT and EuroSCORE. URL: upper reference limit; N: total number of patients; HR: hazard ratio; 95% CI: 95% confidence interval; AUC; area under the Receiver Operator Characteristic curve.

## Data Availability

Related raw or aggregated data related to this article could be made available via the corresponding author upon reasonable request.

## Supplementary Materials

The following supporting information can be downloaded at: https://www.mdpi.com/article/10.3390/jcm12134291/s1.
