# Supplementary material for: Defining Peri-Operative Myocardial Injury during Cardiac Surgery Using High-Sensitivity Troponin T
Source: J Clin Med. 2023 Jun 26;12(13):4291. doi: 10.3390/jcm12134291 (PMC10342425; doi:10.3390/jcm12134291)
Supplement: Supplementary file 1 [file jcm-12-04291-s001.zip › jcm-2413790-supplementary.pdf]

Online Supplemental Appendix

Supplementary Table S1. Performance of hs-TnT elevation at 6, 12, 24, 48 and 72 hours after CABG in predicting 1-year all-cause mortality among patients with EF >50% (N=795)

Time point	X URL	Sensitivity	Specificity	Adjusted AUC (95% CI)*	Adjusted HR (95% CI)*
6h	50	71%	48%	0.701 (0.632-0.770)	1.25 (0.74-2.14)
	70	51%	66%	0.700 (0.629-0.771)	1.31 (0.77-2.25)
	100	46%	82%	0.713 (0.642-0.785)	2.30 (1.30-4.08)
12h	35	85%	33%	0.703 (0.634-0.772)	1.32 (0.70-2.49)
	50	72%	53%	0.713 (0.646-0.779)	1.77 (1.04-3.01)
	70	49%	72%	0.718 (0.653-0.784)	2.02 (1.20-3.39)
24h	35	75%	51%	0.704 (0.635-0.772)	1.41 (0.81-2.46)
	50	62%	69%	0.715 (0.647-0.782)	1.87 (1.08-3.23)
	70	46%	82%	0.714 (0.645-0.783)	1.86 (1.04-3.33)
48h	35	69%	65%	0.730 (0.667-0.793)	2.38 (1.34-4.22)
	50	52%	79%	0.721 (0.655-0.786)	2.01 (1.13-3.56)
	70	39%	88%	0.734 (0.666-0.801)	2.75 (1.50-5.03)
72h	30	65%	64%	0.719 (0.654-0.784)	1.95 (1.09-3.50)
	35	58%	69%	0.716 (0.651-0.781)	1.84 (1.05-3.20)
	50	44%	81%	0.713 (0.644-0.781)	1.85 (1.00-3.41)

*Adjusted for baseline hs-TnT and EuroSCORE

URL: upper reference limit; N: total number of patients; HR: hazard ratio; 95% CI: 95% confidence interval; AUC; area under the Receiver Operator Characteristic curve

Supplementary Table S2. Performance of hs-TnT elevation at 6, 12, 24, 48 and 72 hours after CABG in predicting 1-year all-cause mortality among patients with EF 30-50% (N=239)

Time point	X URL	Sensitivity	Specificity	Adjusted AUC (95% CI)*	Adjusted HR (95% CI)*
6h	50	68%	51%	0.615 (0.465-0.764)	1.72 (0.62-4.76)
	70	54%	68%	0.597 (0.449-0.745)	1.72 (0.64-4.67)
	100	40%	85%	0.635 (0.484-0.786)	2.91 (0.99-8.47)
12h	35	74%	40%	0.594 (0.451-0.736)	1.21 (0.41-3.59)
	50	69%	58%	0.637 (0.490-0.783)	2.08 (0.74-5.81)
	70	55%	74%	0.631 (0.480-0.782)	2.26 (0.81-6.30)
24h	35	66%	55%	0.613 (0.464-0.762)	1.72 (0.61-4.84)
	50	58%	73%	0.626 (0.474-0.778)	2.24 (0.76-6.56)
	70	53%	89%	0.679 (0.525-0.834)	4.39 (1.53-12.59)
48h	35	61%	64%	0.615 (0.459-0.771)	1.89 (0.66-5.42)
	50	55%	80%	0.653 (0.497-0.808)	3.11 (1.02-9.53)
	70	48%	88%	0.692 (0.538-0.847)	5.28 (1.69-16.45)
72h	30	67%	63%	0.626 (0.465-0.787)	2.15 (0.77-6.01)
	35	65%	69%	0.652 (0.491-0.812)	2.99 (1.02-8.74)
	50	51%	83%	0.669 (0.516-0.822)	3.81 (1.24-11.66)

*Adjusted for baseline hs-TnT and EuroSCORE

URL: upper reference limit; N: total number of patients; HR: hazard ratio; 95% CI: 95% confidence interval; AUC; area under the Receiver Operator Characteristic curve

Supplementary Table S3. Performance of hs-TnT elevation at 6, 12, 24, 48 and 72 hours after CABG in predicting 1-year all-cause mortality among patients with EF <30% (N=123)

Time point	X URL	Sensitivity	Specificity	Adjusted AUC (95% CI)*	Adjusted HR (95% CI)*
6h	50	63%	62%	0.731 (0.573-0.889)	1.40 (0.43-4.54)
	70	56%	77%	0.717 (0.557-0.876)	0.96 (0.27-3.44)
	100	29%	92%	0.744 (0.580-0.908)	4.11 (1.08-15.59)
12h	35	89%	51%	0.739 (0.578-0.900)	1.54 (0.46-5.15)
	50	58%	71%	0.726 (0.562-0.889)	1.32 (0.41-4.27)
	70	54%	83%	0.725 (0.567-0.882)	1.18 (0.33-4.21)
24h	35	67%	62%	0.720 (0.558-0.881)	1.15 (0.35-3.74)
	50	57%	80%	0.729 (0.567-0.890)	1.44 (0.42-4.91)
	70	48%	88%	0.716 (0.556-0.876)	0.84 (0.20-3.47)
48h	35	67%	75%	0.752 (0.583-0.920)	2.71 (0.79-9.29)
	50	56%	88%	0.760 (0.598-0.922)	2.85 (0.80-10.10)
	70	41%	93%	0.743 (0.576-0.910)	3.95 (1.12-13.92)
72h	30	83%	73%	0.804 (0.653-0.954)	5.01 (1.31-19.11)
	35	76%	78%	0.772 (0.604-0.941)	3.54 (1.11-11.25)
	50	50%	90%	0.752 (0.583-0.921)	4.65 (1.45-14.91)

*Adjusted for baseline hs-TnT and EuroSCORE

URL: upper reference limit; N: total number of patients; HR: hazard ratio; 95% CI: 95% confidence interval; AUC; area under the Receiver Operator Characteristic curve

<u>Supplementary Figure S1</u>. Unadjusted area under the curve (AUC) for each hs-TnT elevation at 6, 12, 24, 48 and 72 hours after CABG

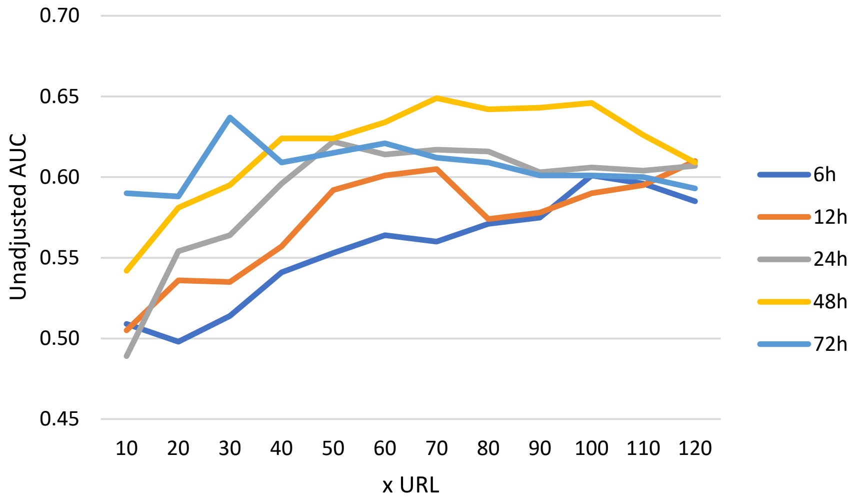

The first peak of the AUC occurred at 100xURL at 6 hours, 70xURL at 12 hours, 50xURL at 24 hours, 70xURL at 48 hours, and 30xURL at 72 hours. Hence, we selected these thresholds. Thresholds <50xURL at 6 hours, ≥100xURL at 12 hours, 24 hours and 48 hours, and ≥70xURL at 72 hours were not shown in the main tables as there were very few outcomes at these thresholds. In addition, we looked at threshold ≥35xURL as the Society for Cardiovascular Angiography and Interventions defined clinically relevant PMI as ≥35xURL elevation in troponin in the presence of additional evidence of coronary ischemia or ≥70xURL elevation by itself.
